# The Emerging Role of Extracellular Vesicle-Associated RNAs in the Multiple Myeloma Microenvironment

**DOI:** 10.3389/fonc.2021.689538

**Published:** 2021-06-21

**Authors:** Jihane Khalife, James F. Sanchez, Flavia Pichiorri

**Affiliations:** ^1^Judy and Bernard Briskin Center for Multiple Myeloma Research, City of Hope, Duarte, CA, United States; ^2^Department of Hematologic Malignancies Translational Science, City of Hope, Duarte, CA, United States

**Keywords:** multiple myeloma, microenvironment, extracellular vesicles, non-coding RNA, biomarker

## Abstract

Multiple myeloma (MM) is a cancer of terminally differentiated plasma cells (PCs) that develop at multiple sites within the bone marrow (BM). MM is treatable but rarely curable because of the frequent emergence of drug resistance and relapse. Increasing evidence indicates that the BM microenvironment plays a major role in supporting MM-PC survival and resistance to therapy. The BM microenvironment is a complex milieu containing hematopoietic cells, stromal cells, endothelial cells, immune cells, osteoclasts and osteoblasts, all contributing to the pathobiology of MM, including PC proliferation, escape from immune surveillance, angiogenesis and bone disease development. Small extracellular vesicles (EVs) are heterogenous lipid structures released by all cell types and mediate local and distal cellular communication. In MM, EVs are key mediators of the cross-talk between PCs and the surrounding microenvironment because of their ability to deliver bioactive cargo molecules such as lipids, mRNAs, non-coding regulatory RNA and proteins. Hence, MM-EVs highly contribute to establish a tumor-supportive BM niche that impacts MM pathogenesis and disease progression. In this review, we will first highlight the effects of RNA-containing, MM-derived EVs on the several cellular compartments within the BM microenvironment that play a role in the different aspects of MM pathology. We will also touch on the prospective use of MM-EV-associated non-coding RNAs as clinical biomarkers in the context of “liquid biopsy” in light of their importance as a promising tool in MM diagnosis, prognosis and prediction of drug resistance.

## Introduction

Multiple myeloma is a hematological malignancy characterized by clonal expansion of plasma cells (PCs) at different sites within the bone marrow (BM). It progresses from monoclonal gammopathy of undetermined significance (MGUS) and smoldering myeloma (SMM). Diagnosis may include the presence of more than 10% of malignant plasma cells in the BM. Despite the introduction over the last two decades of novel treatments such as proteasome inhibitors and immunomodulatory drugs that significantly improved patients’ survival, MM is still incurable, typically with multiple episodes of remission and relapse (1). One of the reasons why MM is highly heterogenous and variable in survival is that its pathogenesis is strongly associated with a BM-tumor supportive environment. MM-PCs highly depend on various components of the BM microenvironment for growth and survival. The dynamic cross-talk between MM-PCs and cells of the BM microenvironment play a crucial role in modifying the oncogenic landscape of the microenvironment in favor of disease occurrence, progression and resistance to treatment (1, 2). The BM microenvironment is a complex milieu composed of a cellular and non-cellular compartment. The cellular compartment contains hematopoietic cells, mesenchymal stromal cells, endothelial cells, immune cells, osteoclasts and osteoblasts, all contributing to the pathobiology of MM, including PC proliferation, escape from immune surveillance, angiogenesis and bone disease development (2).

Extracellular vesicles (EVs) are lipid bilayers that are heterogenous in size and content; they are released by all cell types, both normal and malignant, known to be involved in cell-cell communication at short and long distances (3). EVs were first recognized as a route of cellular waste release (4). However, more studies done on their biogenesis and content have revealed novel findings that have implication in transfer of information in both health and disease (5). In MM, EVs play a pivotal role in mediating a mutual communication between MM-PCs and cells of the tumor microenvironment by transferring bioactive molecules such as lipids, proteins, and regulatory RNAs, all contributing to MM pathobiology (6). Moreover, EVs are abundantly released in the sera of MM patients carrying selective molecular cargoes indicative of disease state (7). Therefore, MM-EVs play an essential role as biomarkers of disease in the context of liquid biopsies, where solid biopsies have limited accessibility.

The RNA cargo of EVs mainly consists of messenger RNA (mRNA) and non-coding RNA (ncRNA) and varies among different biofluids (8) and among vesicles from different cell types (9). In this context, non-coding RNAs (microRNAs, piwi-RNAs, long non-coding RNAs and circular RNAs) were shown to be the most enriched nucleic acid bioactive cargoes in the EVs of cancer patients (10). The biological functions of EV-regulatory RNAs were well identified in solid tumors and were the subject of phase I clinical trials (11–13). However, their role in hematological malignancies is in the process of being defined. In this review, we will focus on discussing the effects of RNA-containing, MM-derived EVs on the cellular compartments within the BM microenvironment that play a crucial role in the various aspects of MM pathology. We will also highlight the important role of circulating EV-non-coding RNAs as diagnostic markers and biomarkers of MM progression and drug resistance in the context of liquid biopsy.

### Extracellular Vesicles: Biogenesis and Trafficking

The International Society for Extracellular Vesicles (ISEV) uses EVs as a general description to all particles, released by all cell types, that lack a nucleus but exhibit lipid bilayers. EVs are classified according to different features, including orders of size, mechanism of biogenesis, cell of origin and cargo content. According to the updated guidelines of Minimal Information for Studies of Extracellular Vesicles 2018 (14), assigning EVs on the basis of their biogenesis pathways or subtype markers remains difficult. Therefore, MISEV18 recommends to name EVs in accordance with their physical characteristics such as size, biochemical composition, or described conditions. Herein, the main classification of EVs will be their order of size, aligned with the latest classification of MISEV2018. Two main subgroups have been defined: i) small EVs; ii) medium/large EVs. Both subgroups contain several subclasses based on cellular origin, morphology, function and biogenesis (14, 15).

- Small EVs (sEVs) represent the smallest EVs. They are less than 100 nm or 200 nm in size. Exosomes are an example of sEVs. They range in size between 30 to 150 nm. They are derived from the endocytic compartment and undergo further maturation to form intraluminal vesicles. They carry a wide variety of cargo molecules including RNAs (mRNAs and non-coding regulatory RNAs) and proteins. EV-encapsulated mRNAs can be translated to proteins by the recipient cells (14, 16). Retrovirus-like particles are another example of small EVs. They range between 90-100 nm in size, they directly bud from the plasma membrane, and they contain retroviral particles, cytoskeleton proteins, or plasma membrane components (17).
- Medium/large EVs (m/lEVs) >200 nm in size (14). Some types of microvesicles or ectosomes that range in size from 200 to 1000 nm can be considered medium EVs. They are formed by plasma membrane budding. They release their cargoes by remodeling the cortical cytoskeleton. Unlike exosomes, they are not formed in a consistent manner but by specific cell types. Their cargoes consist of RNAs and proteins similar to exosomes. However, microvesicles are the only particles that can additionally encapsulate plasmid DNA (18, 19). Large vesicles reach up to 10,000 nm in size and contain cytoskeletal structures (14, 20, 21). Apoptotic bodies are an example of large vesicles with a diameter greater than 1000 nm. Their name is based on the description of condition of cell of origin (14). They are formed from cleavage of the cytoplasmic membrane upon activation of apoptosis or programmed cell death. They contain both cytosolic components (RNA and protein) and nuclear fragments (22).

EVs are released by all cell types under both physiological conditions and stress responses. They carry specific bioactive molecules (RNA, protein), and their composition is strictly dependent on the parental cells from which they originated. They release their content in the surrounding interstitial space or biofluids (urine, blood), where they are named ‘‘circulating vesicles,’’ or they can be taken up by recipient cells at short or long distances, facilitating cell-cell communication (23). At the destination site, EVs release their cargoes by several mechanisms, including membrane fusion, phagocytosis, receptor-mediated caveolin-clathrin or endocytosis mediated by lipid rafts, enabling specific molecular functions in the recipient cells (24). In recent years, the close association between EVs and tumor cell survival has been a major focus of research. Indeed, tumor cells have been shown to secrete dramatically higher quantities of EVs in the plasma than their healthy counterparts. The majority of EVs secreted by malignant cells contained specific cargo molecules involved in tumor development, further supporting the role of EVs in tumor growth and disease progression (25).

## Characterization of MM-PCs and MM-BM Cells-Derived EVs

Malignant PCs often home at multiple sites within the BM. Because MM extensively relies on the microenvironment for development, EVs are considered essential mediators by which MM cells disrupt BM microenvironment homeostasis in favor of tumor development (26). Although to date, standardized methods for proper isolation and characterization of ultrapure EVs are still lacking (27), a high-throughput technique has been purposely applied to characterize MM-derived EVs for optimal functional analysis of their contents using a minimum amount of starting material (28).

We have used global systemic proteomic analysis to characterize EVs isolated from MM and have identified bioactive proteins involved in multiple biological processes that contribute to MM development. For instance, we have identified elevated levels of CD44, MHC class I and bone marrow stromal antigen 2 (BST-2) in MM-derived EVs, with respect to their healthy counterparts. Moreover, levels of membrane molecules such as CD38, CD44, and CD9 were significantly higher in Ig light chain-positive EVs from MM patients as compared to those isolated from MGUS (29). At the RNA level, Manier et al. reported that small noncoding microRNAs were the most predominant regulatory RNAs differentially expressed in MM patients and cancer-free controls. For instance, a small RNA sequencing of circulating EVs identified 158 differentially expressed exosomal microRNAs (30), among which some were previously identified as oncogenic targets in malignant PCs (31)and some were significantly differentially expressed between high and low-risk MM (32). Importantly, exosomal microRNAs were not only released by malignant PCs but also by cells of the microenvironment to exert a mutual communication between MM cells and the BM microenvironment. Indeed, microarray studies revealed that MM-bone marrow stromal cells (MM-BMSCs) initiated the transfer of EVs carrying functional microRNAs, resulting in MM cell proliferation, survival and migration, whereas EVs isolated from BMSCs of healthy controls contained a different microRNAs profile and instead inhibited MM tumor growth (33, 34). Although studies have specifically demonstrated the involvement of exosomal microRNAs in a cross-talk between MM-PCs and cells of the BM microenvironment (30, 33, 35), an analysis of EV-associated regulatory RNAs on a wider spectrum is yet to be defined. More studies on a larger number of PCs isolated from MM patients using deep exosomal RNA-sequencing are needed to further unravel the contribution of EV-RNAs other than microRNAs in MM pathogenesis. We will review what has been so far demonstrated about the role of EV-associated regulatory RNAs in the cross-talk between MM-PCs and compartments of the BM microenvironment that influence MM pathogenesis, including tumor growth, immune evasion, angiogenesis and bone disease development.

## MM-EV-RNAs and Mesenchymal Stem Cells (MSCs)

Bone marrow stromal cells consist of diverse cell populations including BM mesenchymal stem cells (MSCs), fibroblasts, osteoblasts, chondrocytes and adipocytes. They play an essential role in supporting hematopoietic cell development and maturation. In cancer, stromal cells acquire a distinct molecular signature that allows them to evolve in concordance with the disease (36). In MM, BMSCs play a crucial role in disease development and progression by either direct cell-cell contact or indirectly through a mutual communication *via* EVs. Such interactions result in the release of pro-tumorigenic factors by BMSCs that contribute to MM pathobiology (37). The content of BM-MSC-derived exosomes from MM patients was analyzed and revealed a major breakthrough in the field of vesicular RNA biology. MM-BM-MSC-EVs carrying low levels of the tumor suppressor *miR-15a* were found to be the cause of deregulation of several signaling pathways, including the Akt pathway. Thus, MM-MSCs-derived EVs were engulfed by MM-PCs and induced the proliferation, survival, and migration of the latter by activating oncogenic factors known to be targets of the Akt pathway (33, 38). These findings highlight the importance of EV-RNAs in supporting MM pathogenesis. Interestingly, a recent study showed that a long non-coding RNA *LINCOO461* that acts as a decoy for miR-*15a* and miR-*16* tumor suppressors was transferred to MM cells *via* MSC-derived EVs, resulting in inhibition of both miRNAs and upregulation of their target anti-apoptotic gene *BCL2*. As a result, EVs enriched in *LINCOO461* inhibited apoptosis of MM cells and induced their proliferation (39).

In line with these findings, another study demonstrated that EVs released by BMSCs of MM patients impacted disease survival and progression. In fact, EVs isolated from BMSCs of MM patients contained a distinct microRNA profile from those isolated from healthy controls. In particular, oncomiRNA *miR-10a* was transferred in EVs at high levels to MM cells, resulting in the proliferation of MM-PCs. In MM cells, *miR-10a* was shown to positively regulate the expression of β-TRC (a key regulator of IκB-α degradation in the NF-κB pathway). Inhibiting β-TRC abrogated miR-*10a*-mediated MM cell proliferation (34). However, a study showed that MSCs isolated from healthy individuals acquired the tumor phenotype and molecular signature of MSCs from MM patients only when cocultured with malignant PCs (40). Thus, whether MM cells first educate stromal cells and modify their homeostasis in favor of PC survival is not well understood. More work has to be done in this regard to clarify whether MM-PCs are the first initiators of a cross-talk *via* EVs. For instance, several studies demonstrated that MM-PCs release high amounts of EVs in circulation as compared to normal PCs. Those EVs have been shown to associate with MM patients’ survival and can serve as useful biomarkers for prediction of disease progression (24). In the context of the BM microenvironment, EVs from MM-PCs were able to promote proliferation of MSCs and their transformation to cancer-associated fibroblasts (CAFs). CAFs are one of the most dominant stromal cells that play a pivotal role in the MM-BM niche. When transformed, CAFs selectively release pro-tumorigenic soluble factors including tolerogenic cytokines, chemokines that support MM-PCs. In a recent study, Cheng et al. showed that MM cells release EVs carrying high amounts of the oncomiRNA *miR-21* and were selectively taken up by BM-MSCs. In BM-MSCs, *miR-21* activated expression and secretion of the growth factors SDF-1, FAP and α-SMA that in turn induced MSC transformation to CAF. As a result, CAFs caused an increase in MM cell proliferation. Moreover, inhibition of *miR-21* in EVs led to a decrease in CAF markers and MM-PC growth (41). In agreement with these findings, another group has observed that MM-EVs were enriched in another oncomiRNA, *miR-146a*, known to play a major role in several types of cancer including MM. MM-EVs with high amounts of *miR-146a* were transferred to MSCs, inducing in the recipient cells an increase in IL-6 secretion, a cytokine known to promote MM cell survival. EV-*miR-146a* also stimulated the expression and release of growth factors known to increase MM-PCs’ viability and migration such as CXCL-1, IP-10, CCL2 and CCL5. This phenomenon was dependent on the NOTCH signaling pathway. In fact, inhibition of NOTCH signaling in MSCs abrogated the *miR-146a*-induced increase in cytokines/chemokines, as well as MM cell growth and migration (42).

Under normal physiological conditions, MSCs differentiate to osteoblasts known as active bone forming cells. However, MSCs exposed to MM-derived EVs have a reduced capacity to differentiate towards bone nodules, which results in a reduction of bone formation. One mechanism of action that induces such a phenomenon was recently demonstrated by Li et al. The research group found that bioactive lncRNA RUNX2-AS1 in MM cells were encapsulated into EVs and transferred to MSCs. lncRNA RUNX2-AS1 is a long non-coding RNA that represses RUNX2, a key transcription factor that is involved in osteogenic differentiation, by disrupting its splicing. Transfer of lncRNA RUNX2-AS1 resulted in decreased differentiation of MSCs, whereas attenuation of lncRUNX2-AS1 in EV-producing MM cells significantly restored the osteogenic potential of MSCs (43).

## MM-EV-RNAs and Osteoclasts/Osteoblasts

MM symptoms include anemia, renal failure and proteinuria. Additionally, one of the major complications associated with MM progression, affecting more than 80% of patients, is osteolytic bone disease. Patients suffer from persistent bone lesions, leading to pain, fractures, mobility issues and neurological defects (44). Bone formation or osteogenesis involves the collaboration of different group of cells and proceeds through several steps. It requires a tight balance between two major cellular types: osteoblasts and osteoclasts. While osteoblasts, originally differentiated from MSCs in the first step of osteogenesis, are actively involved in bone development; osteoclasts, originating from the fusion of multiple precursor cells, are involved in resorption of bones and reshaping (45). In MM, the balance of this tightly coupled process is wholly impaired, resulting in increased osteoclastic activity together with suppression of osteoblastic activity, both leading to progressive disruption of the bone tissue and osteolytic bone disease (40). The pathological processes and molecular mechanisms involved in MM-related bone disease are well defined. Communication between MM-PCs invading the BM and resident bone cells is believed to be the main cause of shifting the balance to reduced osteoblastogenesis, increased osteoclastogenesis and, ultimately, osteolytic lesions. In fact, once MM cells engraft into the BM, they establish a vicious cycle with bone cells. They have been shown to interact with osteoclasts by expressing essential signaling factors such as MIP-1α, RANKL, MMP-13 and Decoy receptor 3 (DcR3), all known to be responsible for increased osteoclast formation (46–48). Concomitantly, MM cells inhibit osteoblast formation by secreting high amounts of Dickkopf-related protein 1 (DKK1), a key soluble factor known to inhibit the Wnt pathway in osteoblast precursors (pre-osteoblasts). Wnt signaling is essential to promote bone morphogenic protein (BMP-2)-mediated differentiation of pre-osteoblasts to mature osteoblasts in the marrow. In addition to increased osteoclastogenesis, DKK1-induced inhibition of Wnt signaling is an underlying mechanism involved in bone loss in MM (49). Moreover, propagation of osteoblastogenesis inhibition among neighboring osteocytes was reported to be due to secretion of high levels of sclerostin by MM-PCs, a DKK1-dependent factor also known to inhibit the Wnt pathway, leading to decreased bone regeneration (50).

In this context, studies have also shown that MM cells secrete factors as cargo in EVs that have dual functions to both inhibit osteoblast development and stimulate osteoclast differentiation and their bone resorption activity. In a recent study, Zhang et al. found that MM-EVs were enriched in molecules that negatively regulate osteogenesis both *in vitro* and *in vivo* and positively correlated with bone lesions in MM patients. By conducting RNA-sequencing, the research group found that a specific regulatory miRNA, miR-103-3p, which plays a crucial role in osteogenesis by targeting the osteoblast developing factor RUNX2, was highly elevated in MM-PC-derived EVs. MM-EVs enriched in miR-103-3p were transferred to BM-MSCs, resulting in impairment of osteoblast differentiation (51). Moreover, another study revealed that miR-129 also played a role in vesicle-mediated bone disease. miR-129 was selectively packaged in EVs that were released by MM-PCs but not by PCs of smoldering myeloma, where bone lesions do not occur. MM-EVs carrying high levels of miR-129 were selectively taken up by MSCs and inhibited the differentiation of the latter to osteoblasts. Mechanistically, miR-129 inhibited the expression of the transcription factor Sp1 and its target alkaline phosphatase, both positive modulators of osteoblastic differentiation (52). Other inhibitors of bone growth contributing to osteolysis were also found to be enriched in EVs shed by MM cells and transferred to MSCs. Among those factors is DKK-1 mRNA. Once in the recipient cells, DKK-1 mRNA is translated to protein and was shown to downregulate the expression of Runx2, Osterix and Collagen 1A1, all involved in osteoblast differentiation (53).

MM-EVs not only inhibit osteoblast differentiation but also support bone break-down by enhancing osteoclast formation. Raimondi et al. were the first to show that MM-derived EVs induced the migration of pre-osteoclasts and increased their proliferation through activation of the chemokine receptor CXCR4. MM-EVs were also shown to induce the differentiation of pre-osteoclasts to multinuclear mature osteoclasts capable of promoting bone resorption (54). Moreover, MM-PCs cells and MM-BMSCs showed to secrete high levels of osteoclast-activating cytokines such as the receptor activator of nuclear factor κB-ligand (RANKL), a key factor and dominant mediator of osteoclast differentiation, activation and survival (55). Additionally, MM-PCs also inhibited the secretion of osteoprotegerin (OG), an important factor involved in osteoblast differentiation. A tight connection between OG and RANKL has been observed. OG acts as a decoy; it antagonizes the biding of RANKL to its receptor, which results in the inhibition of the NK-κB pathway, thereby preserving the integrity of bone mass. Consequently, a balanced RANKL/OG ratio is essential for maintaining proper bone development (56). In MM, the ratio is significantly disrupted favoring RANKL and NF-κB pathway activation, resulting in the transcription of several downstream factors involved in bone lesions. Pitari et al. detailed the molecular mechanism that causes such a disruption. They showed that both PCs and BMSCs isolated from MM patients secrete high levels of the oncomiRNA miR-21 encapsulated in EVs (41, 57). Once transferred to normal BM-MSCs, miR-21 directly targets the 3’UTR of OG, resulting in its degradation and the inhibition of its secretion. Additionally, miR-21 enhances STAT-3 dependent signaling by suppressing PIAS3, the inhibitor of STAT-3, thereby resulting in constitutive STAT3-mediated RANKL gene expression. By both inhibiting OG and activating RANKL, overexpression of miR-21 in the MM microenvironment plays a pivotal role in shifting the balance towards osteoclast proliferation and activation, causing progressive bone resorption (57). Another important osteoclastic factor that is specifically enriched in MM-derived EVs is amphiregulin (AREG). Raimondo et al. reported that EV-AREG shed by MM-PCs were able to induce the differentiation of osteoclasts from their precursors by activating epidermal growth factor receptor and its downstream target, SNAIL. MM-EVs carrying high amounts of AREG not only acted on pre-osteoclasts but were also taken up by human MSCs, leading to increased MM cell adhesion, release of the pro-osteoclastogenic cytokine IL-8 and inhibition of osteoblast differentiation (58).

In addition to the impaired osteoblast/osteoclast balance that is known to significantly contribute to MM-bone disease, studies have demonstrated the essential role of medullary adipocytes in MM- bone resorption (59). Adipocytes are differentiated from MSCs and secrete metabolically active molecules such as adipokines, growth factors and inflammatory activators, all contributors to loss of bone mass (60). In fact, an increase in both the pre-adipocyte and mature adipocyte numbers as well as adipokine secretion were reported to recruit macrophages into the BM microenvironment, a major source of TNF-α. TNF-α induces RANKL activation by adipocytes, which in turn leads to the differentiation of MSCs to osteoclasts (61). Moreover, a cross-talk between adipocytes and osteoblasts was shown to affect the adipocyte/osteoblast balance. For instance, a study revealed that EVs released by adipocytes enriched in adipogenic PPARγ, leptin, CEBPα and CEBPδ mRNAs, as well as the anti-osteoblastic miR-138, miR30c, miR125a, miR-125b, and miR-31 -miRNAs were transferred to BM-MSC-derived osteoblasts, resulting in increased expression of adipocytic transcripts, marrow adiposity and subsequent bone lesions (62).

MM-PCs also altered the balance. More recently, Liu et al. demonstrated that a direct contact between MM-PCs and MSCs shifted MSC differentiation toward adipocytes over osteoblasts. The research group showed that Integrin α4 on the surface of MM cells activated the adhesion molecule VCAM1 on MSCs, leading to inhibition of the E3 ubiquitin ligase MURF1, which resulted in stabilization of the adipocyte transcription factor PPARγ2 (63).

In addition to their detrimental role in MM-related bone disease, adipocytes further enhance MM cell growth by releasing secretory factors used as a source of energy by MM-PCs. Hence, several reports demonstrated that elevated levels of adipokines such as leptin, adipsin, and visfatin promoted MM cell proliferation and resistance to chemotherapy (64–66). Moreover, adipocytes induce homing of MM cells to the BM by secreting chemoattractants such as MCP-1 and SDF-1α (67). Consequently, MM cells in the BM microenvironment adhere to adipocytes that in turn protect MM-PCs from apoptosis-induced chemotherapy (60).

## MM-EV-RNAs and Endothelial Cells

Endothelial cells are important components of the BM microenvironment. They are part of the vascular niche and play an essential role in the formation of new blood vessels from pre-existing vessels, a physiological process named angiogenesis. In cancer, tumor cell proliferation, survival and dissemination are highly dependent on angiogenesis, as cancerous cells require comparatively more nutrients and oxygen from the vasculature. Blocking endothelial cell proliferation and tube formation is a unique therapeutic approach used to prevent growth and spread of malignant cells (68). In MM, BM angiogenesis is a hallmark of disease progression; it is present in the majority of patients requiring treatment and correlates with poor prognosis (69). A study using primary samples revealed that supernatants of MM-PCs induced significantly higher *in vitro* angiogenesis compared to that from normal BM-PCs. Most of the secretory molecules that enhanced blood vessel sprouts are known pro-angiogenic factors, such as vascular endothelial growth factor (VEGF), basic fibroblast growth Factor (bFGF), and angiopoietin-1 (70). In fact, EVs secreted by MM cells contributed to angiogenesis by transferring high amounts of VEGF to endothelial cells, resulting in their proliferation and capillary structure formation (71). The mechanism underlying the binding of MM-EVs to endothelial cells and the delivery of their cargoes has been recently identified. Purushothaman et al. found that MM-EVs expressing high levels of fibronectin on their surface specifically bind to heparin sulfate on endothelial cells, thus mediating MM-EV-BM endothelial cell interaction (72). Moreover, regulatory RNAs have also been shown to act as key factors in EV-mediated BM angiogenesis. A study reported that MM-EVs carrying high levels of the oncomiRNA miR-135b were selectively transferred to BM endothelial cells. In recipient cells, miR-135b directly suppressed its target, hypoxia-inducer factor 1 (FIH-1), a negative regulator of HIF-1α. As a result, under hypoxic conditions, high levels of pro-angiogenic factors, known to be activated *via* the HIF1-α signaling pathway, are secreted by endothelial cells, leading to enhanced endothelial tube formation and angiogenesis both *in vitro* and *in vivo*. Consequently, EVs released by MM-PCs under hypoxic conditions were shown to carry even higher levels of miR-135b, creating a positive feedback loop that leads to extremely low oxygen levels in the MM microenvironment to further sustain BM-angiogenesis (73). In addition to microRNAs, piwi-interacting RNAs were shown in a recent report to be important regulators involved in MM angiogenesis. PiRNA-823 is encapsulated in EVs, accumulates in the peripheral blood of MM patients and positively correlates with late stage disease and poor prognosis (74). It was also found to mediate intercellular communication between MM-PCs and the BM-microenvironment. In particular, MM-derived EVs enriched in piRNA-823 were transferred to endothelial cells causing an increase in proliferation, tube formation and invasion by enhancing the expression of VEGF and IL6 and attenuating apoptosis in the target cells (75).

## MM-EVs and Immune Cells

Immune cells are important defensive cells located in the BM microenvironment of patients with hematological malignancies. The ability of tumor cells to evade immune surveillance is a key factor for disease progression and metastatic dissemination (76). Tumor derived EVs are believed to participate in the cross-talk between malignant and the surrounding immune cells, resulting in suppression of the immune response in order to create a tumor-supportive environment (77). In MM, the cargoes of tumor derived EVs that play a role in repressing the immune defense are not well characterized. However, studies investigated the biological functions of MM-PC- and MM-BMSC-derived EVs on immune cells of the BM microenvironment. Natural killer (NK) cells, which are an essential component of the innate immune system known to exert cytotoxic effects against microbial infections and tumor cells, are the most among immune cells that are affected by MM-EVs. Xiong et al. first demonstrated that MM-PC-derived EVs significantly reduced the cytotoxic activity of NK cells against MM. The effect was mediated *via* transferring of repressor factors that induced a dramatic decline in the expression levels of activating receptors NKp46, NKp30 and NKG2D, known to play an essential role in NK cytotoxicity (78). Moreover, another study showed that, under genotoxic stress, MM cells released high amounts of EVs that were selectively taken up by NK cells, resulting in suppression of the latter’s activity (79). At the molecular level, transfer of the metalloproteinase ADAM10 was believed to be involved in repression of NK cytotoxicity. ADAM10 is known to shed NKG2D receptor ligand MIC. Once shed, MIC becomes soluble and blocks NKG2D by binding to its activating domain (80). Another type of cargo carried by MM-PC-derived EVs that inhibits NK cell function is the MM-associated antigen CD38. The CD38 molecule is either bound to the surface of PCs or exists in a soluble form that acts as an ectoenzyme that converts nucleotides to adenosine. Adenosine binds to purinergic P2 receptors present on the surface of immune cells, leading to their anergic response. MM-PC-derived EVs were shown to be enriched in the soluble form of CD38 and CD38 mRNA; they were transferred to NK cells and caused suppression of cytotoxicity against MM cells (81).

Myeloid derived suppressor cells (MDSCs) are another type of immune cell located in the MM-BM microenvironment that showed to play an essential role in MM progression. MDSCs are a small population of immature myeloid cells that suppress the function of effector T cells, thereby contributing to MM-PC escape from immune surveillance (82). In fact, MM-BMSCs were shown to communicate with MDSCs *via* EVs. EVs released by BMSCs were transferred to MDSCs and induced the proliferation of the latter in a mechanism dependent on STAT3 and STAT1 signaling pathways. As a result, overgrown MDSCs secreted high levels of nitric oxide in the MM-BM microenvironment, causing T cell death and escape of MM-PCs from cytotoxic T cell attack (83).

Other BM immune cells that are key mediators of MM cell survival are tumor associated macrophages (TAMs). Once differentiated from monocytes, macrophages have the plasticity to polarize to either M1 (tumoricidal) or M2 (tumorigenic) macrophages. M1 macrophages are essential in activating tumor immune response by secreting pro-inflammatory cytokines whereas M2 macrophages produce anti-inflammatory cytokines/chemokines that not only suppress the immune response but also contribute to malignant cell proliferation and survival (84). *In vivo*, M2 macrophages are also named tumor associated macrophages (TAM). In particular, the MM-BM microenvironment was found to be extensively infiltrated with TAMs, and their levels were positively correlated with poor prognosis (85). In addition, TAMs promoted MM progression and metastatic dissemination (86). To our knowledge, we were the first to show the involvement of EVs in a mutual communication between MM-PCs and monocytes/macrophages, resulting in a shift of the oncogenic landscape of the BM microenvironment. In a recent study, we found that MM-PCs carrying a chromosome 13 aberration (Del13) released abundant amounts of EVs that were deficient in the tumor suppressor miR-16. MM-derived EVs were transferred to monocytes, leading to differentiation of the latter to M2 tumor-supportive macrophages. However, EVs released by PCs without Del13 did not affect monocyte differentiation. At the molecular level, we demonstrated that miR-16 targeted the IKKα/β complex of the NF-κB canonical pathway, leading to lowered expression of key growth factors and cytokines implicated in M2 differentiation (IL-10, IL-8, TNF-α). Consequently, in the absence of the transfer of high levels of miR-16 to monocytes, enhanced NF-κB activity as well as increased expression and production of tolerogenic cytokines/chemokines essential for M2 macrophage polarization occurs (35).

### Role of EV-Non-Coding Regulatory RNAs as Biomarkers in MM

MM is a hematological malignancy where diagnosis, prognosis and prediction of disease progression are somewhat challenging because of the lack of powerful monitoring tools for estimating disease outcome and differentiating MM from other neoplasia; therefore, non-invasive biomarkers are urgently need to better monitor the disease and predict treatment response (87). Circulating free regulatory non-coding RNAs (ctrfRNAs) in patients’ plasma have proved to be useful predictive tools to monitor disease development. However, the major limitations to use ctrfRNAs as a liquid biopsy is their instability and short half lives in biological fluids (88). EVs encapsulating regulatory RNAs have accordingly been shown to be a comparatively strong non-invasive biomarker platform because of their high abundance in biofluids and their ability to protect their cargoes from nuclease and protease degradation (30, 89). In fact, patients with MM release significantly higher levels of EV-carrying markers such as CD38 and CD138 in their plasma with respect to healthy controls (7). In particular, circulating microRNAs carried by EVs were differentially expressed between MM patients and healthy controls. RNA sequencing on the aforementioned circulating exosomes revealed that, among a large exosomal microRNA panel, only EV-let7b and EV-miR-18a were associated with disease prognosis. Patients with severe disease had low levels of EV-let7b and EV-miR-18a in circulation, and their serum levels negatively correlated with progression-free survival and overall survival (30). Moreover, measuring EV-microRNA levels is not only a useful tool to monitor MM progression but could also be applicable to discriminate between MM and its precursor states. For instance, high levels of EV-miR-129 were detected in the BM and sera of patients with MM but not in patients with smoldering myeloma. miR-129 targets several genes involved in osteoblast differentiation, and its overexpression is associated with bone diseases; therefore, circulating EV-miR129 levels could be a suitable indicator of MM bone disease development (52), a complication not observed in MGUS or smoldering myeloma.

We showed that, other than its tumor suppressive role in communicating between MM-PCs cells and cells of the BM microenvironment, EV-miR-16 is among the few microRNAs encapsulated in EVs that were associated with MM prognosis in patients treated with proteasome inhibitor-based therapy. Patients who released high levels of EV-miR-16 in their sera had significantly longer five years overall survival compared to those with lower levels of EV-miR-16 (90). In agreement with these findings, Zhang et al. conducted an exosome-associated miRNA expression pattern on patients resistant to bortezomib. The research group found that exosomal miR-16, miR-15a, miR-20a, and miR-17 were strongly downregulated in circulation and were associated with drug resistance (91). A majority of MM patients develop resistance to bortezomib during the course of treatment; thus, circulating EV-miR-16 and -miR15a could be of important use as biomarkers of treatment response. In addition to microRNAs, lncRNAs are other regulatory RNAs delivered by EVs and are released in large levels in circulation. A recent study showed that the lncRNA LNC00461, which binds miR-15a/16, was highly expressed in MM exosomes (39). Monitoring EV-LNC00461 levels in the sera of MM patients could be another approach to predict MM progression and resistance to bortezomib treatment. Other than their role in MM prognostication, EVs carrying lncRNAs can also be usedp to distinguish malignancies with monoclonal gammopathies. For instance, the lncRNA content of EVs released in the plasma of MGUS and MM patients were shown to be different. However, among 84 lncRNAs secreted, only one exosomal lncRNA, PRINS (psoriasis susceptibility-related RNA gene induced by stress), was found to be dysregulated in MM and MGUS patients compared to healthy controls. Levels of exosomal PRINS positively correlated with clinical parameters such as bone marrow plasma cell infiltrate rate, albumin, creatinine, and lactate dehydrogenase levels (92). These findings emphasize the potential diagnostic role of circulating EV-PRINS in discriminating monoclonal gammopathies from healthy subjects. The role of MM-EVs as biomarkers of disease and their biological functions are summarized in **Table 1**.

**Table 1:** Overview of the role of MM-EV-associated ncRNAs as biomarkers of disease and their biological functions.

Type of RNA	Cargo	Biomarkers’ Use	Biological function	References
microRNA	↓ Let-7b	Diagnosis Prognosis	Target Myc Inhibit PC proliferation	(30, 93)
	↓ miR-18a	Diagnosis Prognosis	Target HIF-1α, IRF2 activate M1 macrophages	(30, 94, 95)
	↑ miR-129	Diagnosis	Target SP1 Inhibit osteoblast differentiation	(52)
	↓ miR-15a, ↓ miR-16	Prognosis Drug resistance	Target Akt3, BCL-2, IKKβ Inhibit PC proliferation Inhibit M2 macrophage	(35, 96)
	↓ miR-17 ↓ miR-20a	Prognosis Drug resistance	Target Myc Induce PC apoptosis	(91)
LncRNA	↑ LNC00461	NI Possible prognosis, drug resistance	Sponge for miR-15a/16 Increase PC proliferation	(39)
	↑ PRINS	Diagnosis	Regulate the anti-apoptotic factor G1P3	(92, 97)
			Inhibit apoptosis	

MM, Multiple myeloma; EV, Extracellular vesicle; ncRNA, Non-coding RNA; PC, Plasma cell ; ↓, Low levels;­­ ↑, high levels; LncRNA, Long non-coding RNA; NI, Not identified.

## Discussion

**Figure 1:**
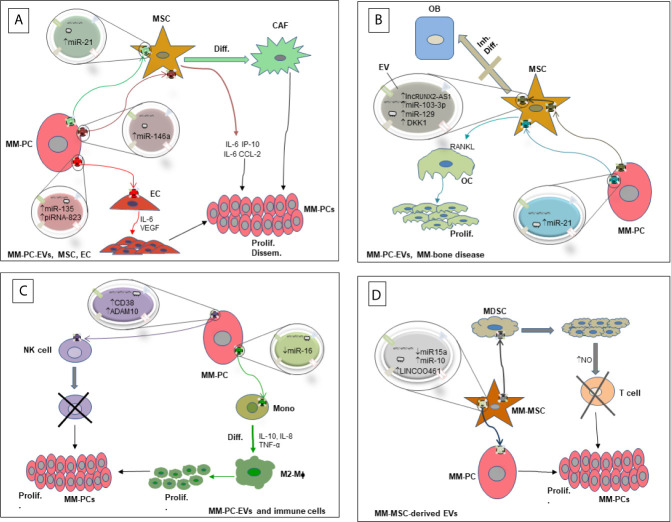
Diagram showing the interaction of MM-PCs with several components of the BM-microenvironment *via* RNA-containing EVs and their biological effects. **(A)** Interaction of MM-PC-derived EVs with MSCs and ECs. **(B)** Interaction of MM-PC-derived EVs with OCs and OBs leading to MM-bone disease. **(C)** Interaction of MM-PC-derived EVs with immune cells. **(D)** Interaction of MM-MSC-derived EVs with MM-PCs and other cellular components of the MM microenvironment. EV, Extracellular Vesicle; MM-PC, Multiple myeloma plasma cell; MDSC, Myeloid derived suppressor cell; MM-MSC, Multiple myeloma derived mesenchymal stromal cell; MSC, mesenchymal stromal cell; CAF, Cancer associated fibroblast; OB, Osteoblast; OC, Osteoclast; EC, Endothelial cell; Mono, Monocyte; M2-Mϕ, M2 macrophage; NK cell, Natural killer cell; RANKL, receptor activator of nuclear factor κB-ligand; NO, Nitric oxide; Diff., Differentiation; Inh. Diff., Inhibition of differentiation; prolif., Proliferation; Dissem., Dissemination; ↑, High levels; ↓, Low levels.

We have shown clear evidence of the involvement of EVs in re-educating the BM microenvironment in favor of MM survival and progression. In particular, we discussed the involvement of ncRNAs contained in EVs derived from MM-PCs in the several cellular compartments of the BM microenvironment that highly impact MM-PC proliferation and escape from immune surveillance, increase angiogenesis and worsen the severity of MM-associated bone disease. Although studies have confirmed the contribution of EV-RNAs in a mutual communication between MM-PCs and different cell types in the BM-microenvironment, some of which are reviewed above (**Figure 1**), the mechanisms involved in cell target specificity and route of cellular uptake are yet to be fully identified. For instance, a recent study shed light on the identity of the surface molecules on MM-EVs that played a role in directing EVs towards endothelial cells (72). Similar work should be done to unravel the full EV receptor molecular spectrum responsible for guiding EVs to specific target cells in the MM-BM microenvironment. Moreover, MM-EV-associated ncRNAs are highly abundant in circulation, and their signature is different from those released by non-cancerous cells (7); therefore, as their analysis is novel, they are being considered as distinct and feasible non-invasive biomarkers of disease occurrence, progression and resistance to therapy. Because EV contents reflect the cells where they originated from (18), other than being useful prognostic and diagnostic tools, EVs containing RNAs released by MM cells or cells of the microenvironment are considered tumor specific EVs, rendering them potential therapeutic targets. In this context, suppression of EV biogenesis or secretion could be a plausible therapeutic approach in MM. However, it could result in both favorable and unfavorable consequences because of the essential role in maintaining normal physiological responses by EVs that are released by healthy cells (98). Hence, applying innovative technologies that simultaneously characterize and specifically detect tumor-associated EVs on the basis of molecular identity of their cargoes is needed. Thanks to modern high-throughput technology and development of multi-omics analysis techniques, targeting tumor specific EVs in MM could become possible.

## Author Contributions

JK, JS, and FP wrote the manuscript. All authors contributed to the article and approved the submitted version.

## Conflict of Interest

The authors declare that the research was conducted in the absence of any commercial or financial relationships that could be construed as a potential conflict of interest.
