# Erratum: The Emerging Role of Extracellular Vesicle-Associated RNAs in the Multiple Myeloma Microenvironment

**DOI:** 10.3389/fonc.2021.740028

**Published:** 2021-07-27

**Authors:** Frontiers Production Office

**Affiliations:** Frontiers Media SA, Lausanne, Switzerland

**Keywords:** multiple myeloma, microenvironment, extracellular vesicles, non-coding RNA, biomarker

Due to a production error, the article was published as an Original Research article. The correct article type is Review. The publisher apologizes for this mistake.

The original version of this article has been updated.

